# The Dietary Guidelines for Americans (2020–2025): Pulses, Dietary Fiber, and Chronic Disease Risk—A Call for Clarity and Action

**DOI:** 10.3390/nu13114034

**Published:** 2021-11-12

**Authors:** Henry J. Thompson

**Affiliations:** Cancer Prevention Laboratory, Colorado State University, Fort Collins, CO 80523, USA; henry.thompson@colostate.edu; Tel.: +1-970-491-7748; Fax: +1-970-491-3542

The 2020–2025 Dietary Guidelines for Americans (DGA) were recently released [1]. Many changes were made in the process that led to the publication of the DGA. These changes were intended to increase transparency and to ensure that the guidelines are based on the best available scientific evidence. As stated in the report, the DGA is a tool to assist professionals and policymakers in the translation of the Dietary Recommended Intakes (DRIs) [2] into dietary patterns that promote human health and well-being and that reduce disease risk. Additionally, noted in the report is that the formulation of the DGA is an evolutionary process and that essentially all individuals can benefit from changes in the choice of foods that constitute the dietary patterns that they follow. While the DGA are intended for use by professionals, an effort was made to make the information provided accessible to the consumer. Many notable changes were made in the DGA. The purpose of this editorial, and of the short communication that accompanies it [3], is to contribute to the evolutionary process underlying DGA formulation so that the groundwork for the 2025–2030 DGA is developed over the next few years.

The DGA is a translational instrument creating a bridge from the DRIs to both healthy dietary patterns and the policies that support their implementation and dissemination. While these guidelines have the implicit intent of reducing the likelihood of inadequate intake of recognized nutrients, there is also an explicit focus on reducing the risk of chronic diseases that account for almost 70% of mortality both in the United States and around the world and that are primary drivers of healthcare costs [4]. The DGA’s acknowledgement of dietary fiber intake as a public health concern is based on evidence that inadequate dietary fiber intake increases chronic disease risk [5,6], an effect that is at least in part mediated by the gut-associated microbiome [7]. The magnitude of the dietary fiber gap, i.e., the difference between actual and recommended intake levels, is approximately a 50% shortfall, and in the U.S. more than 90% of women and 97% of men do not meet the recommended intakes for dietary fiber [1]. Accordingly, the highlighting of dietary fiber as a dietary component of public health concerns is warranted, even though fiber is not considered an essential nutrient.

With the importance of the dietary fiber gap in mind, the DGA panel took an important step in identifying pulses, i.e., grain legumes such as chickpeas, dry beans, dry peas, and lentils, as a distinct type of food legume. However, as detailed in [3], there is a lack of clarity in the use of terminology in the DGA and in the suggested use of pulses. Consequently, the DGA fails to capitalize on the distinctive high-fiber nutrient profile of pulses. Population and clinical data support the value of pulses in reducing chronic disease risk [8,9]. Thus, a specific definition of the term pulse is provided in [3], along with a simple classification schema for legumes—with a focus on pulses—derived from an understanding of seed development. In addition, the nutrient content of pulses is contrasted with other DGA food categories based on nutrient content per 100 kilocalorie (kcal) edible portion. Basing food comparisons on 100 kcal edible portions is consistent with the DGA’s focus on eating foods with high nutrient density. This approach also resonates with efforts to reduce obesity risk via controlling caloric intake. Based on these analyses [3], the argument is advanced that in the next version of the DGA, consideration should be given to how best to recognize pulses relative to their content of dietary fiber. This recommendation is consistent with calls for precision nutrition for the individual, particularly as it relates to the surge of interest in dietary fiber, gut health, and the prevention of chronic diseases [10].

The DGA also recognized that essentially all individuals can improve the food choices that comprise their own personal dietary patterns. Given this recommendation, it is essential to underscore the importance of encouraging these changes to be in the selection of whole foods rather than ingredient-based foods for the reasons detailed in [7,11]. Specifically, evidence was presented that ingredients in many convenience foods negatively impact the composition and function of the gut microbiome and consequently human health and disease risk. In this regard, the culinary versality of pulses was not considered in the DGA, in particular the fact that pulses are most frequently consumed as whole foods [12]. Moreover, while pulse consumption in the U.S. has been reported to be quite low [9], an argument can be made that this is due to a lack of clarity about the definition and classification of pulses and the absence of greater specificity in evidence-based recommendations regarding health-promoting levels of consumption. Because of the culinary versatility of pulses, there is a remarkable opportunity to “re-introduce” this food category and to eliminate the dietary fiber gap. This is important for chronic disease risk reduction given the recognized health benefits of dietary fiber derived from whole foods rather than from the fractionated ingredients into which foods can be processed [13], as well as the numerous health benefits that have been associated with pulses [14,15,16].

While not specifically emphasized, the 2020–2025 DGA identify an immediate focus for future outreach and dissemination: eliminating the dietary fiber gap via increased pulse consumption. The full development of this opportunity will require basic and applied research to delineate the contributions that increased pulse consumption can make to advance public health and reduce chronic disease risk, for instance by impacting the gut-associated microbiome through increased dietary fiber intake.
